# Total knee arthroplasty without patella resurfacing leads to worse results in patients with patellafemoral osteoarthritis Iwano Stages 3–4: a study based on arthroplasty registry data

**DOI:** 10.1007/s00167-023-07387-y

**Published:** 2023-04-04

**Authors:** Paul Nardelli, Sabrina Neururer, Kerstin Gruber, David Wippel, Nadine Kogler, Sebastian Ender, Hermann Leitner, Benedikt Koller, Martin Fischer, Dietmar Dammerer, Michael Liebensteiner

**Affiliations:** 1 Department of Orthopaedics and Traumatology, Medical University of Innsbruck, Anichstrasse 35, 6020 Innsbruck, Austria; 2 Department of Clinical Epidemiology, Tyrolean Federal Institute for Integrated Care, Tirol Kliniken GmbH, Innsbruck, Austria; 3 Department of Orthopaedics, St. Vinzenz Hospital, Zams, Austria; 4 Department of Orthopaedics and Traumatology, Krems University Hospital, Krems, Austria; 5 Orthopädie Knie & Fuß im Zentrum, Innsbruck, Austria

**Keywords:** Patella resurfacing, Patellofemoral osteoarthritis, Total knee arthroplasty, Knee osteoarthritis, Iwano score, Patella, Kneecap

## Abstract

**Purpose:**

To determine whether the preoperative degree of degeneration of the patellofemoral joint really affects the outcome of total knee arthroplasty (TKA) surgery without patella resurfacing and thus to establish a parameter that might serve as a guiding factor to decide whether or not to perform retropatellar resurfacing. It was hypothesized that patients with preoperative mild patellofemoral osteoarthritis (Iwano Stages 0–2) would significantly differ from patients with preoperative severe patellofemoral osteoarthritis (Iwano Stages 3–4) in terms of patient-reported outcome (Hypothesis 1) and revision rates/survival (Hypothesis 2) after TKA without patella resurfacing.

**Methods:**

Application of a retrospective–comparative design on the basis of Arthroplasty Registry data that included patients with primary TKA without patella resurfacing. Patients were allocated to the following groups based on preoperative radiographic stage of patellofemoral joint degeneration: (a) mild patellofemoral osteoarthritis (Iwano Stage ≤ 2) and (b) severe patellofemoral osteoarthritis (Iwano Stages 3–4). The Western Ontario and MacMaster Universities Osteoarthritis Index (WOMAC) score was assessed preoperative and 1 year postoperative (0: best, 100 worst). In addition, implant survival was calculated from the Arthroplasty Registry data.

**Results:**

In 1209 primary TKA without patella resurfacing, postoperative WOMAC total and WOMAC subscores did not differ significantly between groups, but potentially suffered from type 2 error. Three-year survival was 97.4% and 92.5% in patients with preoperative mild and severe patellofemoral osteoarthritis, respectively (*p* = 0.002). Five-year survival was 95.8% vs. 91.4% (*p* = 0.033) and 10-year survival was 93.3% vs. 88.6% (*p* = 0.033), respectively.

**Conclusions:**

From the study findings, it is concluded that patients with preoperative severe patellofemoral osteoarthritis have significantly higher risks for reoperation than do those with preoperative mild patellofemoral osteoarthritis—when treated with TKA without patella resurfacing. Hence, it is recommended that patella resurfacing be applied in patients with severe Iwano Stage 3 or 4 patellofemoral osteoarthritis during TKA.

**Level of evidence:**

III, Retrospective comparative.

## Introduction

In the history of orthopaedics the question whether patellae should be resurfaced during primary total knee arthroplasty (TKA) is frequently debated. Nevertheless, no consensus has yet been reached. A literature search was conducted to identify only the systematic reviews and meta-analyses of randomized controlled trials [4, 6, 7, 10, 15, 16, 20, 21]. The findings were incongruent in terms of patient-reported outcomes. However, the publications were somewhat congruent in that non-resurfacing led to higher reoperation rates [4, 6, 7, 10, 15, 16, 20, 21]. Therefore, on the basis of those eight publications no clear conclusions can be drawn in terms of a general recommendation on how to always treat a patella during TKA.

An attempt to adopt a more individual approach addresses the idea of individualizing that decision. Factors that can influence a surgeon to resurface or not resurface the patella could be age, intraoperative degeneration of the patella, shape match between prosthetic trochlea and native patella undersurface, rheumatoid arthritis, frontal plane femoral component position, preoperative degeneration of the patellofemoral joint and many more [2, 9, 11, 13, 14, 18].

Also, amongst the individualized recommendations there seems to be no consensus. Taken together, previous research provides no consensus on whether patellae should be resurfaced *in *general, nor is there any consensus on when patellae should be resurfaced on an individualized basis [2, 3].

Therefore, the aim of this study was to examine whether the preoperative degree of degeneration of the patellofemoral joint really affects the outcome of TKA surgery without patella resurfacing and in this way to establish a parameter that might serve as a guiding factor.

It was hypothesized that patients with preoperative mild patellofemoral osteoarthritis (Iwano Stages 0–2) would significantly differ from patients with preoperative severe patellofemoral osteoarthritis (Iwano Stages 3–4) in terms of patient-reported outcome (Hypothesis 1, primary outcome) and implant survival (Hypothesis 2, secondary outcome) after TKA without patella resurfacing.

## Methods

**Fig. 1:**
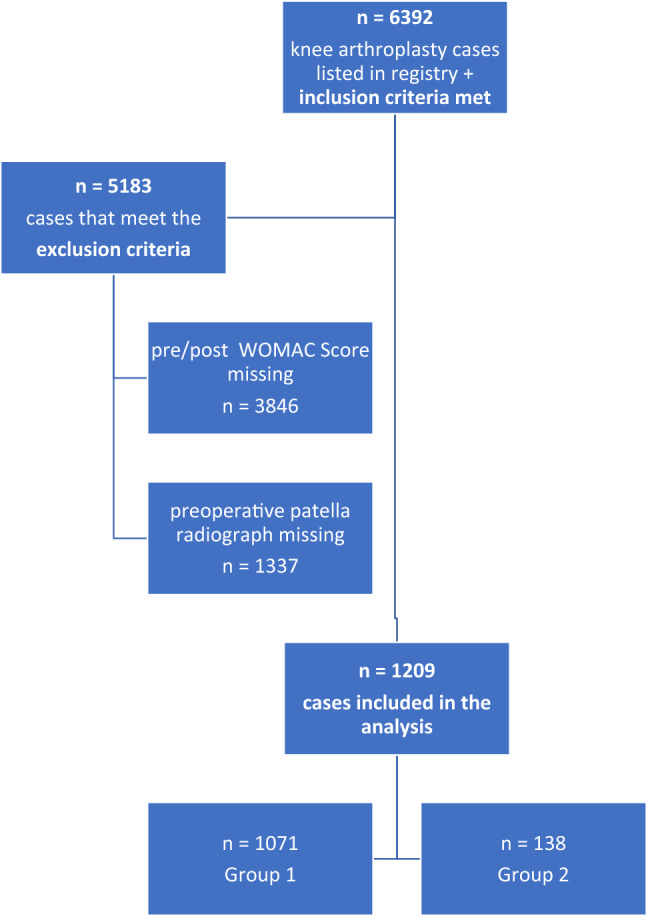
Flowchart of included and excluded patients

A retrospective, comparative study design was applied. Data were extracted from the Arthroplasty Registry of Tyrol. All patients listed in the registry who had undergone primary total knee arthroplasty without patella resurfacing between 2003 and 2019 with study consent were considered for inclusion. Excluded were (a) patients with missing preoperative knee score outcome, (b) patients with missing postoperative knee score outcome (c) or both pre- and postoperative knee score outcome missing and (d) patients with missing preoperative axial patella radiographs from 1 year preoperative (Fig. 1).

In the remaining cases the axial patella radiographs were analyzed. If more than one axial patella radiograph existed, the one closer to the date of surgery was taken for analysis. On the basis of that radiograph the severity of patellofemoral joint degeneration was staged according to the Iwano classification [12]. For further analyses patients were allocated to the following groups based on the Iwano Stages: (1) mild patellofemoral osteoarthritis (Iwano Stage ≤ 2) and (2) severe patellofemoral osteoarthritis (Iwano Stages 3–4). For interrater and intrarater reproducibility correlation coefficients of > 0.9 were determined.

It was hypothesized that patients with preoperative mild patellofemoral osteoarthritis (Iwano Stages 0–2) would significantly differ from patients with preoperative severe patellofemoral osteoarthritis (Iwano Stages 3–4) in terms of patient-reported outcome (Hypothesis 1, primary outcome) and revision rates/survival (Hypothesis 2, secondary outcome) after TKA without patella resurfacing.

For patient-reported outcome measurement the Western Ontario and MacMaster Universities Osteoarthritis Index (WOMAC) questionnaire [1] was available as part of the Arthroplasty Registry data [19]. The questionnaire was completed the day before surgery and again 1 year after surgery, 0% denoting the best and 100% the worst response.

With regard to our secondary hypothesis, we estimated cumulative revision-free survival from date of surgery until date of revision, date of death or end of follow-up (31 Dec 2019), whichever occurred first, by applying the Kaplan–Meier method. Three-, five- and ten-year-implant survival were calculated.

Before starting the retrospective data analysis and identifying potential patients approval was obtained from the Ethics Committee of the Medical University of Innsbruck. (Approval code: 1230/2020).

Data analysis was performed with SPSS, Version 27 (IBM Corp. Released 2020. IBM SPSS Statistics for Windows, Version 27.0, Armonk, NY: IBM Corp.) and with Stata, Version 13 (StataCorp LP, 4905 Lakeway Drive, College Station, TX 77845, USA). Data were not normally distributed, as indicated by the Kolmogorov–Smirnov test. As descriptive values medians and interquartile ranges were determined. Mann–Whitney *U* tests were applied to test for significant differences between groups regarding the WOMAC total score and the WOMAC subscores. Differences in survival curves were tested using the log-rank test.

## Results

Of 6392 patients originally identified, 5183 patients had to be excluded due to the above-mentioned exclusion criteria (Fig. 1). Of the remaining 1209 patients the participants’ characteristics are detailed in Table 1.

**Table 1:** Patient demographics with allocation to Group 1 (mild patellofemoral osteoarthritis) or Group 2 (severe patellofemoral osteoarthritis)

	Total	Group 1	Group 2
N	1209 (100%)	1071 (88.6%)	138 (11.4%)
Age	70 (63–75)	70 (62–75)	72 (66–76)
BMI	28.34 (25.39–32.03)	28.34 (25.32–31.99)	28.09 (25.64–32.37)
IWANO-score			
0	22 (1.8%)	22 (2.1%)	–
1	606 (50.1%)	606 (56.6%)	–
2	443 (36.7%)	443 (41.4%)	–
3	87 (7.2%)	–	88 (63.8%)
4	50 (4.1%)	–	50 (36.2%)
Group			
1	1071 (88.6%)	–	–
2	138 (11.4%)	–	–
Sex			
Female	753 (62.3%)	669 (62.5%)	84 (60.9%)
Male	456 (37.7%)	402 (37.5%)	54 (39.1%)

For parameters age and BMI (body mass index) median ± IQR (interquartile range) are presented, for the other parameters absolute and relative frequencies are presented

Regarding Hypothesis 1, 1 year postoperative the WOMAC total was 11.7 and 13.8 in patients with preoperative mild and severe patellofemoral osteoarthritis, respectively (*p* = 0.247). WOMAC subscale data are detailed in Table 2.

**Table 2̄:** Patient-reported outcome in terms of WOMAC total and WOMAC subscales at preoperative and postoperative points in time

	Group 1			Group 2			*p* value	Power
	Median	IQR		Median	IQR			
Pre								
WOMAC total	53	40	67	51	35	64	n.s	0.42
WOMAC pain	52	36	66	46	32	60	**0.011**	0.81
WOMAC stiffness	55	35	75	55	35	75	n.s	0.07
WOMAC function	54	39	68	50	36	63	n.s	0.46
Post								
WOMAC total	12	5	27	14	6	30	n.s	0.11
WOMAC pain	8	2	20	10	2	20	n.s	0.05
WOMAC stiffness	15	5	30	20	10	35	n.s	0.28
WOMAC function	12	4	28	14	4	28	n.s	0.07
Difference pre–post	36	18	51	29	13	50	n.s	0.53

**Fig. 2:**
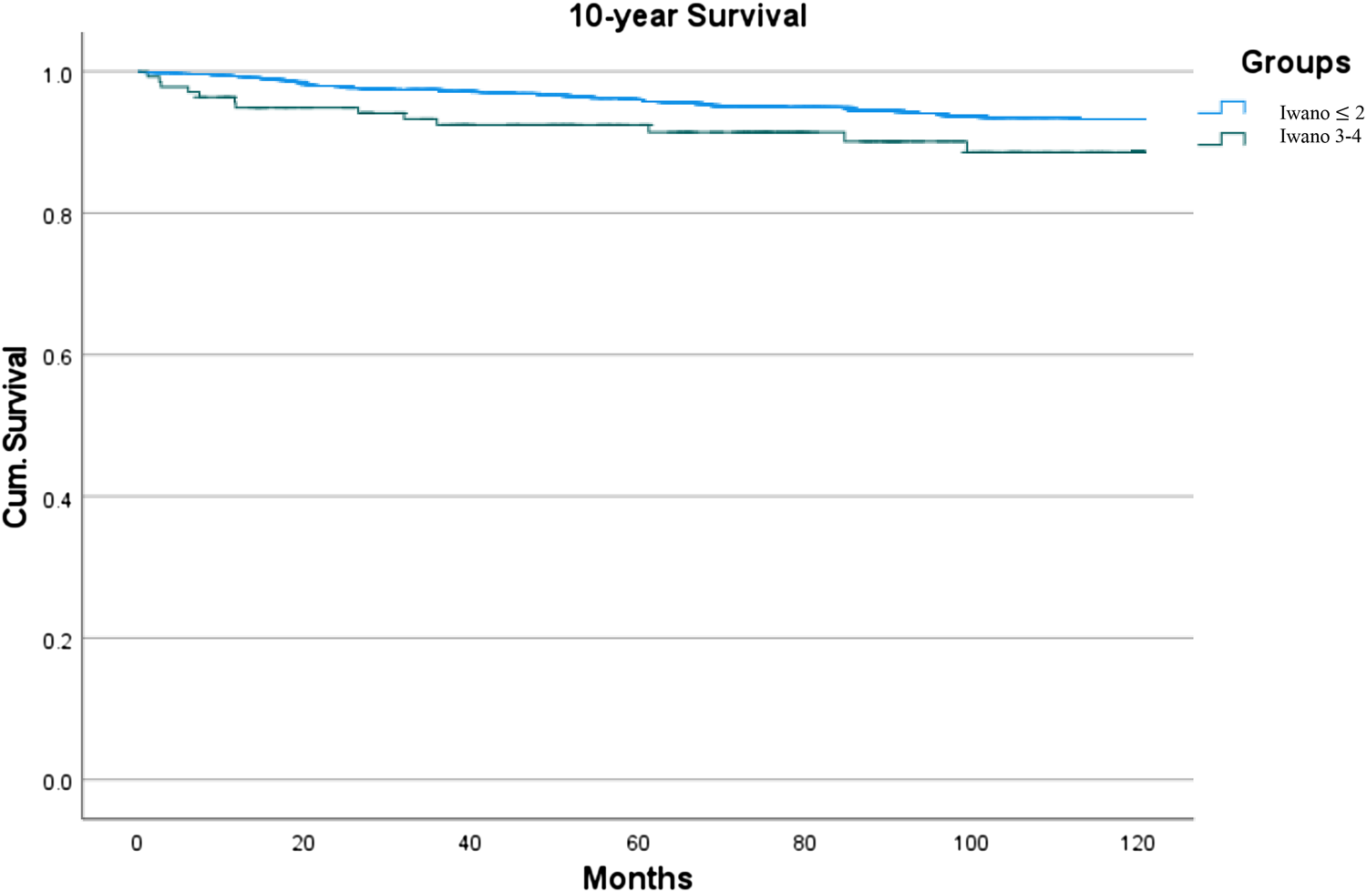
Revision-free survival in patients with mild patellofemoral osteoarthritis (Group 1) and severe patellofemoral osteoarthritis (Group 2) after TKA without patella resurfacing

Regarding Hypothesis 2, the 3-year (revision-free) survival as reported by the Arthroplasty Registry was 97.4% and 92.5% in patients with preoperative mild and severe patellofemoral osteoarthritis, respectively (*p* = 0.002). Five-year survival was 95.8% vs. 91.4% (*p* = 0.033) and 10-year survival was 93.3% vs. 88.6% (*p* = 0.033) (Table 3 and Fig. 2). Follow-up was 89 months (mean, SD 48).

**Table 3:** Revision-free implant survival 3-year, 5-year and 10-year post-surgery in patients with preoperative mild and severe patellofemoral osteoarthritis

Survival	Group 1	Group 2	*p* value	
3 years	97.4%	92.5%	**0.002**	
5 years	95.8%	91.4%	**0.033**	
10 years	93.3%	88.6%	**0.033**	
Median survival (total)	178.83 months	165.88 months	**0.050**	**0.99 Power**

## Discussion

One of the most important findings is that with regard to the WOMAC score there were small inter-group differences of unclear statistical significance. The difference in the parameters ‘postoperative WOMAC stiffness’ and ‘pre-to-postoperative gain in WOMAC total’ was *p* < 0.1 with power values of 28% and 53%. Hence, it may be assumed that differences in WOMAC scores between groups were present, but were not sufficiently detected by the statistics (statistical type 2 error, Hypothesis 1). Moreover, the revision-free survival rate was significantly poorer in patients with severe patellofemoral osteoarthritis when treated with TKA without patella resurfacing—poorer than in those with mild patellofemoral osteoarthritis (Hypothesis 2). On the basis of those findings the authors recommend that patients with preoperatively severe patellofemoral osteoarthritis (Iwano Stages 3 and 4) undergo patella resurfacing during TKA.

In an effort to compare our findings with previous research Cho et al. already investigated whether the preoperative severity of patellofemoral osteoarthritis affected the outcome of TKA without patella resurfacing [5]. The authors reported no significant differences between mild (Stage 0–1 Iwano) and moderate to severe (Stage 2–4 Iwano) cases according to WOMAC and Hospital for Special Surgery scores. From their findings the authors concluded that good results may be achieved with patella non-resurfacing, even in patients with severe patellofemoral osteoarthritis. The gross differences to the findings made in the current study may be explained by differences in the applied methods. First, the current study included far more patients (approx. 1200 vs. approx. 450). Second, the method of allocating patients to study groups according to their Iwano stage was not the same. Most importantly, Cho et al. analyzed postoperative patellofemoral tracking parameters, which may be regarded as surrogate parameters. Instead, the current study calculated the revision-free survival rate, which is regarded as a more robust outcome.

Also Feng et al. investigated the same topic and retrospectively analyzed the data from 167 patients [8]. Preoperative severity of patellofemoral osteoarthritis was again determined according to Iwano (Stage 0–1 was defined as mild and Stage 2–4 as moderate to severe). Several knee scores were applied. The authors determined no significant differences between the groups. The findings made by Feng et al. contradict the findings made in the current study. These differences may be attributed to the differences in numbers of patients and to the differences in the outcome parameters.

Schmidt et al. investigated preoperative severity of patellofemoral osteoarthritis in 193 individuals undergoing TKA without patella resurfacing according to Kellgren–Lawrence and according to the OARSI system [17]. The authors determined the Knee Society Score, global satisfaction, physical activity and the reoperation rate. The authors reported that patients with more severe preoperative osteoarthritis (lateral-sided) benefitted even more from the surgical procedure than did those with less severe osteoarthritis, as determined from the patient-reported outcomes. Reoperation rate was not affected by the stage of preoperative patellofemoral joint degeneration. Again, the differences to the current study are striking, but may be explained by the method differences. Schmidt et al. determined the location of the patellofemoral osteoarthritis in addition to the severity. Moreover, there were substantial differences in the methods regarding the patient-reported outcomes and the numbers of participants.

As stated above, the authors of the current study recommend patella resurfacing at least in patients with severe patellofemoral osteoarthritis. The authors are well aware that an additional surgical step during TKA may also bear additional risks. However, on the basis of the higher revision rates in the subgroup of patients with severe patellofemoral osteoarthritis those risks seem to be outweighed by the benefits. Clearly, thorough surgical training in how to perform patella resurfacing is indispensable. Typical errors like (a) introducing a too high overall patella thickness, (b) malalignment of the patella osteotomy, (c) implant overhang and (d) patella implant malrotation should be avoided.

The following potential limitations of the current study are acknowledged. First, the two sole outcome parameters that were available from the Arthroplasty Registry (WOMAC and revision-free survival) were susceptible not only to patellofemoral problems. In other words, although there was poorer revision-free survival in the group with more severe patellofemoral osteoarthritis, those revisions may not necessarily have been related to patellofemoral reasons.

An additional comparison of ‘reasons for revision’ did not reveal any differences between the two groups. Second, this was a retrospective study with the typical weaknesses associated with such studies: selection bias, information bias, inability to investigate parameters other than those previously collected during clinical routine, reliance on data collected by others etc. Third, although previously suggested [22], we did not succeed in collecting physical activity data and health-related quality-of-life data in conjunction with the knee-specific WOMAC data. Fourth, as mentioned above, our study must be regarded as underpowered with respect to the knee score outcome. However, no further patients would have been available with both complete WOMAC and x ray data. Thus, a-priori sample size calculation would not have solved that problem. Fifth, the retrospective approach based on an arthroplasty registry prevented us from determining how the non-resurfaced patellae were treated intra-operatively (circumpatellar electrocautery, osteophyte removal, neglected etc.). Regarding surgical technique, there was no standardization as the data originated from the state-run Arthroplasty Registry. It has to be assumed that a broad variety of surgical techniques was applied across the different hospitals.

It is regarded as a strength of our study that it employed the by far largest number of patients and the longest follow-up period (10 years). Moreover, the applied outcome parameters were well-established parameters and were regarded as having high data quality as they were derived from the state-run Arthroplasty Registry.

The study findings are deemed to have high clinical relevance. As meta-analyses of the last 10 years draw no clear general conclusion on the question whether or not to resurface patellae during TKA, the question may be addressed in a more individualized way. The findings made in the current study reveal that at least a small subgroup of patients (approx. 5–10%) is potentially better treated with additional patella resurfacing. This additional procedure brings an increase in implant costs and surgical risks, but appears to be beneficial and, therefore, justified in that subgroup of patients in the day-by-day clinical work.

## Conclusions

From the study findings it is concluded that patients with preoperative severe patellofemoral osteoarthritis have significantly higher risks for reoperation than do those with preoperative mild patellofemoral osteoarthritis—when treated with TKA without patella resurfacing. Hence, it is recommended that patella resurfacing be performed in patients with severe Iwano Stage 3 or 4 patellofemoral osteoarthritis during TKA.

## Data Availability

The data that support the findings of this study can only be made available from the corresponding author upon reasonable request.

## References

[1] Bellamy N, Buchanan WW, Goldsmith CH, Campbell J, Stitt LW (1988). Validation study of WOMAC: a health status instrument for measuring clinically important patient relevant outcomes to antirheumatic drug therapy in patients with osteoarthritis of the hip or knee. J Rheumatol.

[2] Burnett RS, Bourne RB (2004). Indications for patellar resurfacing in total knee arthroplasty. Instr Course Lect.

[3] Calvisi V, Camillieri G, Lupparelli S (2009). Resurfacing versus nonresurfacing the patella in total knee arthroplasty: a critical appraisal of the available evidence. Arch Orthop Trauma Surg.

[4] Chen K, Dai X, Li L, Chen Z, Cui H, Lv S (2021). Patellar resurfacing versus nonresurfacing in total knee arthroplasty: an updated meta-analysis of randomized controlled trials. J Orthop Surg Res.

[5] Cho WJ, Bin SI, Kim JM, Lee BS, Sohn DW, Kwon YH (2018). Total knee arthroplasty with patellar retention: the severity of patellofemoral osteoarthritis did not affect the clinical and radiographic outcomes. J Arthroplasty.

[6] Choi KY, In Y, Kim MS, Sohn S, Koh IJ (2022). Is the patient aware of the difference between resurfaced and nonresurfaced patella after bilateral total knee arthroplasty? A systematic review of simultaneous bilateral randomized trials. Knee Surg Relat Res.

[7] Delgado-González A, Morales-Viaji JJ, Arteaga-Hernández JG, Larrosa-Arranz Á, Criado-Albillos G, Martin-Rodríguez ADP (2022). To resurface or not to resurface the patella in total knee arthroplasty, that is the question: a meta-analysis of randomized controlled trials. Medicina (Kaunas).

[8] Feng X, Fan C, Wang F (2021). The impact of severity of patellofemoral osteoarthritis on the patient-reported outcomes of total knee arthroplasty with patellar retention: a retrospective comparative study. Acta Orthop Traumatol Turc.

[9] Fleaca SR, Mohor CI, Dura H, Chicea R, Mohor C, Boicean A (2022). Effect of patella resurfacing on functional outcome and revision rate in primary total knee arthroplasty (Review). Exp Ther Med.

[10] Fu Y, Wang G, Fu Q (2011). Patellar resurfacing in total knee arthroplasty for osteoarthritis: a meta-analysis. Knee Surg Sports Traumatol Arthrosc.

[11] Hsu RW (2006). The management of the patella in total knee arthroplasty. Chang Gung Med J.

[12] Iwano T, Kurosawa H, Tokuyama H, Hoshikawa Y (1990). Roentgenographic and clinical findings of patellofemoral osteoarthrosis. With special reference to its relationship to femorotibial osteoarthrosis and etiologic factors. Clin Orthop Relat Res.

[13] Matz J, Lanting BA, Howard JL (2019). Understanding the patellofemoral joint in total knee arthroplasty. Can J Surg.

[14] McConaghy K, Derr T, Molloy RM, Klika AK, Kurtz S, Piuzzi NS (2021). Patellar management during total knee arthroplasty: a review. EFORT Open Rev.

[15] Pavlou G, Meyer C, Leonidou A, As-Sultany M, West R, Tsiridis E (2011). Patellar resurfacing in total knee arthroplasty: does design matter? A meta-analysis of 7075 cases. J Bone Joint Surg Am.

[16] Pilling RW, Moulder E, Allgar V, Messner J, Sun Z, Mohsen A (2012). Patellar resurfacing in primary total knee replacement: a meta-analysis. J Bone Joint Surg Am.

[17] Schmidt GJ, Farooq H, Deckard ER, Meneghini RM (2012). Osteoarthritic severity in unresurfaced patellae does not adversely affect patient-reported outcomes in contemporary primary TKA. J Am Acad Orthop Surg Glob Res Rev.

[18] Slevin O, Schmid FA, Schiapparelli FF, Rasch H, Amsler F, Hirschmann MT (2017). Coronal femoral TKA position significantly influences in vivo patellar loading in unresurfaced patellae after primary total knee arthroplasty. Knee Surg Sports Traumatol Arthrosc.

[19] Stucki G, Meier D, Stucki S, Michel BA, Tyndall AG, Dick W (1996). Evaluation of a German version of WOMAC (Western Ontario and McMaster Universities) arthrosis index. Z Rheumatol.

[20] Tang XB, Wang J, Dong PL, Zhou R (2018). A meta-analysis of patellar replacement in total knee arthroplasty for patients with knee osteoarthritis. J Arthroplasty.

[21] Teel AJ, Esposito JG, Lanting BA, Howard JL, Schemitsch EH (2019). Patellar resurfacing in primary total knee arthroplasty: a meta-analysis of randomized controlled trials. J Arthroplasty.

[22] Wright RW (2009). Knee injury outcomes measures. J Am Acad Orthop Surg.

